# 

**Published:** 1846-08

**Authors:** 


### CONTENTS

OF NO. II.

---

### ORIGINAL COMMUNICATIONS.

### ESSAYS AND CASES.

ART. I.—An Account of an Epidemic Bilious Fever, which prevailed in Indiana in 1845. By THOS. W. FRY, M.D., of Crawfordsville, Ia. - - - - - 93
ART. II.—Observations on the Topography, Climate, and Disease of Tipton county, Tennessee. By R. B. HARPER, M.D., of Portersville, Tenn. - - - 107
ART. III.—A Case of False Aneurism of the Brachial Artery, on which an action was brought for Malpractice, with recovery of damages. Reported by E. D. FOREE, M.D., of New Castle, Ky. - - - - 122
ART. IV.—Notes on Medical Matters and Medical Men in London. By DAVID W. YANDELL, M.D. - 127

### REVIEWS.

ART. V.—Fevers: their Diagnosis, Pathology, and Treatment. Prepared and edited, with large additions, from the Essays on Fever in Tweedie's Library of Practical Medicine. By MEREDITH CLYMER, M.D. Professor of the Principles and Practice of Medicine in Franklin Medical College of Philadelphia; Consulting Physician to the Philadelphia Hospital; Fellow of the College of Physicians, etc., etc. - - 135
ART. VI.—Proceedings of the Ohio Medical Convention held at Columbus, May 12, 1846. - - - - 146

Art. VII.—Dictionary of Practical Medicine, comprising General Pathology, the Nature and Treatment of Diseases, Morbid Structures, &c. By James Copland, M.D., F.R.S. Edited with additions. By Charles A. Lee, M.D. - - - - - - 152

SELECTIONS FROM AMERICAN AND FOREIGN JOURNALS.

On the Treatment of Aneurism by Compression. - - 153
New test for Prussic Acid. - - - - - - - 156
On certain Pathological conditions of Milk as the cause of disease in Infants. - - - - - - - 157
A Case of Asphyxia from Drowning. - - - - 163
Perforation of the Membrana Tympani for Cure of Deafness. 164
Prize Essay of the Louisiana Medico-Chirurgical Society. - 164
On the Uses of the Lobelia Inflata. - - - - - - 165
Morbid Effects of Tight-Lacing. - - - - - - 166
The Endermic Application of the Sulphate of Quinine. - 172
Trismus Nascentium. - - - - - - - 173
The age at which Insanity is most Prevalent. - - - 176

ORIGINAL INTELLIGENCE.

Medical Society of Tennessee. - - - - - - 177
Professor Bartlett. - - - - - - - - - 178
University of Louisville. - - - - - - - - 179
The Weather.—Health of Louisville. - - - - - 179
East Tennessee Medical Society. - - - - - - 180
Connecticut Medical Society. - - - - - - - 181
Dr. Forbes and Homœopathy. - - - - - - 181
Dunglison's Medical Dictionary. - - - - - - 182
Facts from Correspondents. - - - - - - - 183

